# Synthesis of Symmetrical Tetrameric Conjugates of the Radiolanthanide Chelator DOTPI for Application in Endoradiotherapy by Means of Click Chemistry

**DOI:** 10.3389/fchem.2018.00107

**Published:** 2018-04-10

**Authors:** Alexander Wurzer, Adrienn Vágner, Dávid Horváth, Flóra Fellegi, Hans-Jürgen Wester, Ferenc K. Kálmán, Johannes Notni

**Affiliations:** ^1^Pharmaceutical Radiochemistry, Technische Universität München, Munich, Germany; ^2^Department of Inorganic and Analytical Chemistry, University of Debrecen, Debrecen, Hungary

**Keywords:** Huisgen-reaction, potentiometry, spectrophotometry, phosphinate, radiopharmaceuticals, endoradiotherapy, prostate-specific membrane antigen, theranostics

## Abstract

Due to its 4 carbonic acid groups being available for bioconjugation, the cyclen tetraphosphinate chelator DOTPI, 1,4,7,10-tetraazacyclododecane-1,4,7, 10-tetrakis[methylene(2-carboxyethylphosphinic acid)], represents an ideal scaffold for synthesis of tetrameric bioconjugates for labeling with radiolanthanides, to be applied as endoradiotherapeuticals. We optimized a protocol for bio-orthogonal DOTPI conjugation via Cu(I)-catalyzed Huisgen-cycloaddition of terminal azides and alkynes (CuAAC), based on the building block DOTPI(azide)_4_. A detailed investigation of kinetic properties of Cu(II)-DOTPI complexes aimed at optimization of removal of DOTPI-bound copper by transchelation. Protonation and equilibrium properties of Ca(II)-, Zn(II), and Cu(II)-complexes of DOTPI and its tetra-cyclohexylamide DOTPI(Chx)_4_ (a model for DOTPI conjugates) as well as kinetic inertness (transchelation challenge in the presence of 20 to 40-fold excess of EDTA) were investigated by pH-potentiometry and spectrophotometry. Similar stability constants of Ca^II^-, Zn^II^, and Cu^II^-complexes of DOTPI (log*K*_(CaL)_ = 8.65, log*K*_(ZnL_ = 15.40, log*K*_(CuL)_ = 20.30) and DOTPI(Chx)_4_ (log*K*_(CaL)_ = 8.99, log*K*_(ZnL)_ = 15.13, log*K*_(CuL)_ = 20.42) were found. Transchelation of Cu(II)-complexes occurs via proton-assisted dissociation, whereafter released Cu(II) is scavenged by EDTA. The corresponding dissociation rates [*k*_d_ = 25 × 10^−7^ and 5 × 10^−7^ s^−1^ for Cu(DOTPI) and Cu(DOTPI(Chx)_4_), respectively, at pH 4 and 298 K] indicate that conjugation increases the kinetic inertness by a factor of 5. However, demetallation is completed within 4.5 and 7.2 h at pH 2 and 25°C, respectively, indicating that Cu(II) removal after formation of CuAAC can be achieved in an uncomplicated manner by addition of excess H_4_EDTA. For proof-of-principle, tetrameric DOTPI conjugates of the prostate-specific membrane antigen (PSMA) targeting motif Lys-urea-Glu (KuE) were synthesized via CuAAC as well as dibenzo-azacyclooctine (DBCO) based, strain-promoted click chemistry (SPAAC), which were labeled with Lu-177 and subsequently evaluated *in vitro* and in SCID mice bearing subcutaneous LNCaP tumor (PSMA+ human prostate carcinoma) xenografts. High affinities (3.4 and 1.4 nM, respectively) and persistent tumor uptakes (approx. 3.5% 24 h after injection) confirm suitability of DOTPI-based tetramers for application in targeted radionuclide therapy.

## Introduction

Endoradiotherapy (also termed molecular radiotherapy, radioligand therapy or, if addressing peptide receptors, peptide receptor radionuclide therapy (PRRT) or, if involving alpha-emitting nuclides, targeted alpha therapy) (Oyen et al., 2007) refers to the internal application of radionuclides for therapeutic purposes, above all, for curing cancer. In the respective disease management schemes, radiotherapeutics represent the natural complements to imaging tracers. In a tandem application of both types of agents, referred to as “theranostics,” one targeting mechanism is exploited for delivery of different sorts of radionuclides to tissues, either for diagnostic purposes, that is, with the intention to localize lesions by means of external detection of emitted gamma photons, or to achieve a therapeutic effect via local absorption of particle (i.e., alpha- or beta) radiation. The corresponding radiopharmaceuticals are frequently based on peptides, peptidomimetics, enzyme inhibitors, or similar molecules capable of recognizing a specific cell surface receptor, membrane-bound enzyme, ion channel, or comparable target. The bioactive structures are often decorated with a chelate ligand for kinetically inert complexation of a metal ion radionuclide (Wadas et al., 2010). At present, radiotherapeutics most frequently rely on lanthanide(III)- or chemically related ions, such as ^177^Lu, ^90^Y, ^225^Ac, or ^213^Bi (Notni and Wester, 2018). Recognizing malignant cells by overexpression of above-mentioned surface markers, the radiolabeled bioconjugates deliver these beta- or alpha-emitting isotopes in the lesion, resulting in a local irradiation which kills malignant cells.

**Figure 1:**
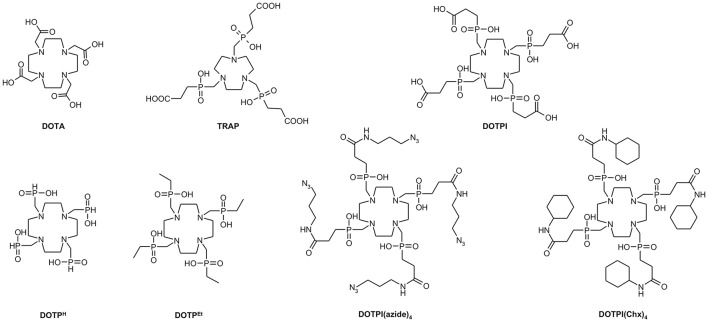
Structures of chelate ligands discussed in the text.

In this context, the chelator DOTA (Figure 1) plays an important role (Stasiuk and Long, 2013) since it forms stable and sufficiently inert complexes with virtually all relevant metal ions. DOTA is usually attached to e.g., peptides via amide formation on one side arm, resulting in derivatives of DOTA-monoamide which is the actual chelator structure in such conjugates (commonly dubbed “DOTA peptides”). However, a likewise functionalization of more than one acetic acid side arm of DOTA yields conjugates whose metal complexes either lack kinetic inertness (e.g., in case of the positron emitter ^68^Ga^III^) or show very slow formation kinetics (namely, with lanthanide(III) ions) (Baranyai et al., 2007; Pasha et al., 2007), limiting practical applicability in radiopharmacy. Conjugates comprising more than one biomolecule (multimers) are nevertheless desirable because a multitude of similar bioactive structure elements in a single framework usually results in enhanced target affinities, and sometimes increases uptake of the respective radiolabeled compounds in target tissues (Maschauer et al., 2017).

For this purpose, we recently designed the DOTA-analog tetraphosphinate chelator DOTPI (Figure 1) featuring four terminal carboxylic acid moieties, which are not required for metal ion complexation and, therefore, are available for bioconjugation (Šimeček et al., 2013). However, similar to the observations made for its smaller congener TRAP (1,4,7-triazacyclononane-1,4,7-tris[methylene(2-carboxyethylphosphinic acid)]) (Notni et al., 2014), amide functionalization of these carboxylates using standard peptide coupling reagents is sometimes complicated by instability of the active ester intermediates (Baranyai et al., 2015). Similar to TRAP, functionalization of DOTPI via “click chemistry,” (Cu^I^-catalyzed alkyne-azide cycloaddition, CuAAC) (Meldal and Tornøe, 2008) employing respective DOTPI-derivatives decorated with “clickable” functional groups, such as DOTPI(azide)_4_ (Figure 1) (Wurzer et al., 2018), appeared to be a valuable alternative. Since the presence of ionic Cu inevitably results in copper complexes of the desired conjugates wherein the Cu ion blocks the coordination site intended for accommodation of the radiometal, removal of chelator-bound Cu, ultimately being transformed to oxidation state +2 during workup, is mandatory to restore the radiolabeling properties. As discussed previously, demetalation with sulfide or cyanide frequently causes problems, while proton-assisted dissociation, promoted by excess of a competing chelator, should be widely applicable and compatible with many synthetic tasks (Notni and Wester, 2016). Against this background, we investigated the metal coordination properties of DOTPI and a model conjugate thereof, DOTPI(Chx)_4_ (Figure 1), with a particular emphasis on Cu^II^ complexes, in order to facilitate optimization of respective demetallation protocols and to support application of CuAAC for elaboration of DOTPI-based multimers.

## Results

In order to enhance legibility, charge, and protonation state of ligands and complexes is indicated only where necessary for comprehension and where possible without ambiguity, e.g., in case it is referred to a single, well defined species and not to a mixture, as is mostly the case due to protonation equilibria. The animal experiments were conducted in accordance with the German Animal Welfare Act (Tierschutzgesetz), and an ethics approval was obtained from the responsible authority (Regierung von Oberbayern).

### Solution thermodynamics

Protonation schemes of cyclens bearing four *N*-substitutents with additional donors (often referred to as DOTA-like ligands) have been thoroughly investigated in the past (Desreux et al., 1981; Bianchi et al., 2000; Takács et al., 2014). Table 1 displays the respective data for DOTPI in comparison to DOTA (Baranyai et al., 2010) and the most closely related tetraphosphinate analogs, DOTP^H^ (Bazakas and Lukeš, 1995; Kotková et al., 2009) and DOTP^Et^ (Lázár et al., 1991) (Figure 1). According to previous literature, it is assumed that the first and second protonation of all chelators under investigation occur at two opposite ring nitrogen atoms. The subsequent protonation steps most likely take place at protonation sites located at the side arms (if available, at the carboxylate groups), due to greater charge separation and lower electrostatic repulsion between the distant protonated donor atoms. Protonations of the phosphinate moieties, characterized by the log$K_{7}^{\text{H}}$ and log$K_{8}^{\text{H}}$ for DOTPI and log$K_{3}^{\text{H}}$ for DOTPI(Chx)_4_, occur only in the acidic pH region (Rohovec et al., 2000), which is why these phosphinate ligands are generally capable of metal complexation and rapid radiolabeling at much lower pH values than chelators with acetic acid side arms, such as DOTA.

**Table 1:** Protonation constants of DOTPI(Chx)_4_, DOTPI, DOTP^H^, DOTP^Et^, and DOTA at 25°C (for structures see Figure 1).

	**DOTPI(Chx)_4_**	**DOTPI**		**DOTP^H^**	**DOTP^Et^**	**DOTA**
***I***	**0.15 M NaCl**	**0.15 M NaCl**	**0.1 M Me_4_NCl (Šimeček et al., 2013)**	**0.1 M KNO_3_**	**0.1 M KNO_3_**	**0.15 M NaCl**
log$K_{1}^{\text{H}}$	**9.89 (1)**	**10.27 (1)**	**11.58**	**10.41, 10.58**	**10.94**	**9.14**
log$K_{2}^{\text{H}}$	**7.73 (1)**	**8.42 (1)**	**8.90**	**6.83, 6.93**	**8.24**	**9.21**
log$K_{3}^{\text{H}}$	3.34 (1)	5.45 (2)	5.65	1.97, 1.90	3.71	4.48
log$K_{4}^{\text{H}}$	–	4.78 (2)	4.98	–	–	4.03
log$K_{5}^{\text{H}}$	–	4.42 (2)	4.61	–	–	1.99
log$K_{6}^{\text{H}}$	–	3.88 (2)	4.07	–	–	1.58
log$K_{7}^{\text{H}}$	–	2.83 (2)	3.12	–	–	–
log$K_{8}^{\text{H}}$	–	1.17 (2)	1.31	–	–	–
Σlog$K_{\text{i}}^{\text{H}}$	20.96	22.69	24.91	19.21, 19.41	22.89	30.43

*Equivalent protonations, occuring for DOTPI and DOTPI(Chx)_4_ at the first and second phosphinate oxygen, are denoted by log$K_{7}^{\text{H}}$/log$K_{8}^{\text{H}}$ and log$K_{3}^{\text{H}}$/log$K_{4}^{\text{H}}$, respectively*.

The equilibrium properties of chelates based on the 1,4,7,10-tetraazacyclododecane-1,4,7,10-tetrakis(methylenephosphinic acid) scaffold depend on the electronic properties of the substituents on the phosphorus atoms (Kotková et al., 2009). log$K_{1}^{\text{H}}$ and log$K_{2}^{\text{H}}$ values for DOTPI(Chx)_4_ are somewhat lower than those of DOTPI, while this lower basicity of the ring nitrogens might be explained by the presence of the more electronegative amide substituents on the pendant arms. However, the lower log$K_{1}^{\text{H}}$ value measured for DOTA in 0.15 M NaCl has another reason; it is explained by the formation of relatively stable [Na(DOTA)]^3−^ complex (log*K*_NaL_ = 4.38) (Chaves et al., 1992). Our data indicate that DOTPI forms a similar NaL complex, entailing a lower log$K_{1}^{\text{H}}$ value in presence of 0.15 M NaCl than in 0.1 M Me_4_NCl (Šimeček et al., 2013).

Total basicity of ligands (Σlog$K_{\text{i}}^{\text{H}}$, Table 1) generally correlates with thermodynamic stability constants (*K*_ML_) of their metal complexes, while in order to obtain chemically meaningful values, the log$K_{\text{i}}^{\text{H}}$ values of the distant carboxylate groups were not considered for calculation of Σlog$K_{\text{i}}^{\text{H}}$ value of DOTPI because they are not involved in metal ion coordination. That being said, it is not surprising that the log*K*_ML_ value of [Ca(DOTA)], [Zn(DOTA)], and [Cu(DOTA)] complexes is about 2–5 log*K* unit higher than phosphinic acid analogs, because DOTA shows a significantly higher total basicity (Table 2).

**Table 2:** Stability constants (log*K*_ML_) for non-protonated DOTPI(Chx)_4_, DOTPI, DOTP^H^, DOTP^Et^, and DOTA complexes formed with divalent metals at 25°C, determined by UV/Vis spectroscopy (a) or potentiometry (b).

	**DOTPI (Chx)_4_**	**DOTPI**		**DOTP^H^(Baranyai et al., 2007)**	**DOTP^Et^(Desreux et al., 1981)**	**DOTA (Takács et al., 2014)**
***I***	**0.15 M NaCl**	**0.15 M NaCl**	**0.1 M Me_4_NCl (Lázár et al., 1991)**	**0.1 M KNO_3_**	**0.1 M KNO_3_**	**0.15 M NaCl**
CuL	**20.42 (1)**^a^	**20.30 (5)**^a^	**23.11**	**18.03**	**19.59**	**21.97 (1)**
ZnL	15.13 (1)^b^	15.40 (2)^b^	18.57	14.60	15.80	17.35 (1)
CaL	8.99 (2)^b^	8.65 (3)^b^	12.48	9.46	9.39	13.84 (1)

*Stepwise protonation constants of the complexes as well as details on experimental procedures and UV/VIS signals used for calculation are given in the Supplementary Information*.

Among the tetraphosphinates, total basicities Σlog$K_{\text{i}}^{\text{H}}$ of DOTPI(Chx)_4_, DOTPI, and DOTP^Et^ ligands are similar but about 1–3 orders of magnitude higher than that of DOTP^H^ which, as expected, is reflected by similar log*K*_CuL_ and log*K*_ZnL_ values for Cu^II^ and Zn^II^ complexes of the *P*-substitued ligands but somewhat lower stabilities for those of DOTP^H^. Thermodynamic stabilities of the Ca^II^ complexes are, however, similar for all phosphinate ligands. This might be explained by differences in the preferred coordination number of these divalent metal ions (Cu^II^ and Zn^II^: 6, Ca^II^: 6–8) owing to their different size (Cu^II^: 73 p.m., Zn^II^: 74 p.m., Ca^II^: 100 p.m.), entailing different structures for Cu^II^ and Zn^II^ complexes as observed for Ca^II^ complexes. Based on available structural data (Riesen et al., 1986), it is assumed that the smaller cations are bound in an N_4_O_2_ coordination environment involving only two side arm oxygens, whereas Ca^II^ is surrounded by a N_4_O_4_ donor set involving all side arms. Hence, side arm basicity and protonation presumably affects the complexation equilibria in a different manner.

Formation of several protonated Cu(H_x_L) species at pH < 7 has been confirmed by pH-potentiometric and spectrophotometric studies of solutions containing Cu^II^ and DOTPI(Chx)_4_ or DOTPI, respectively, while both ligands form Cu^II^ complexes already in acidic solution (pH 1.5 and lower). Since molar absorptivities of the protonated Cu(H_x_DOTPI) and non-protonated Cu(DOTPI) species were found to be essentially equal, it can be assumed that stepwise protonation of Cu(DOTPI) starts with the non-coordinating carboxylates, resulting in Cu(HL), Cu(H_2_L), Cu(H_3_L), and Cu(H_4_L). Two further protonations at comparable pH are observed for Cu^II^ complexes of both DOTPI(Chx)_4_ and DOTPI, while it appears most plausible that they occur on phosphinate oxygens because two of these moieties do not coordinate to Cu^II^ and can thus be protonated.

### Kinetic inertness and transchelation

In order to provide the necessary information for optimization of demetallation protocols, we investigated the pH-dependent kinetics of the transchelation of Cu^II^ from CuDOTPI and CuDOTPI(Chx)_4_ complexes to ethylenediamine-tetraacetic acid (EDTA). Reactions were monitored by UV spectrophotometry at the absorption bands of Cu(DOTPI) and Cu(DOTPI(Chx)_4_) over a pH range of 1.5–4.5, using a 20- and 40-fold excess of EDTA (see Supplementary information).

**Figure 2:**
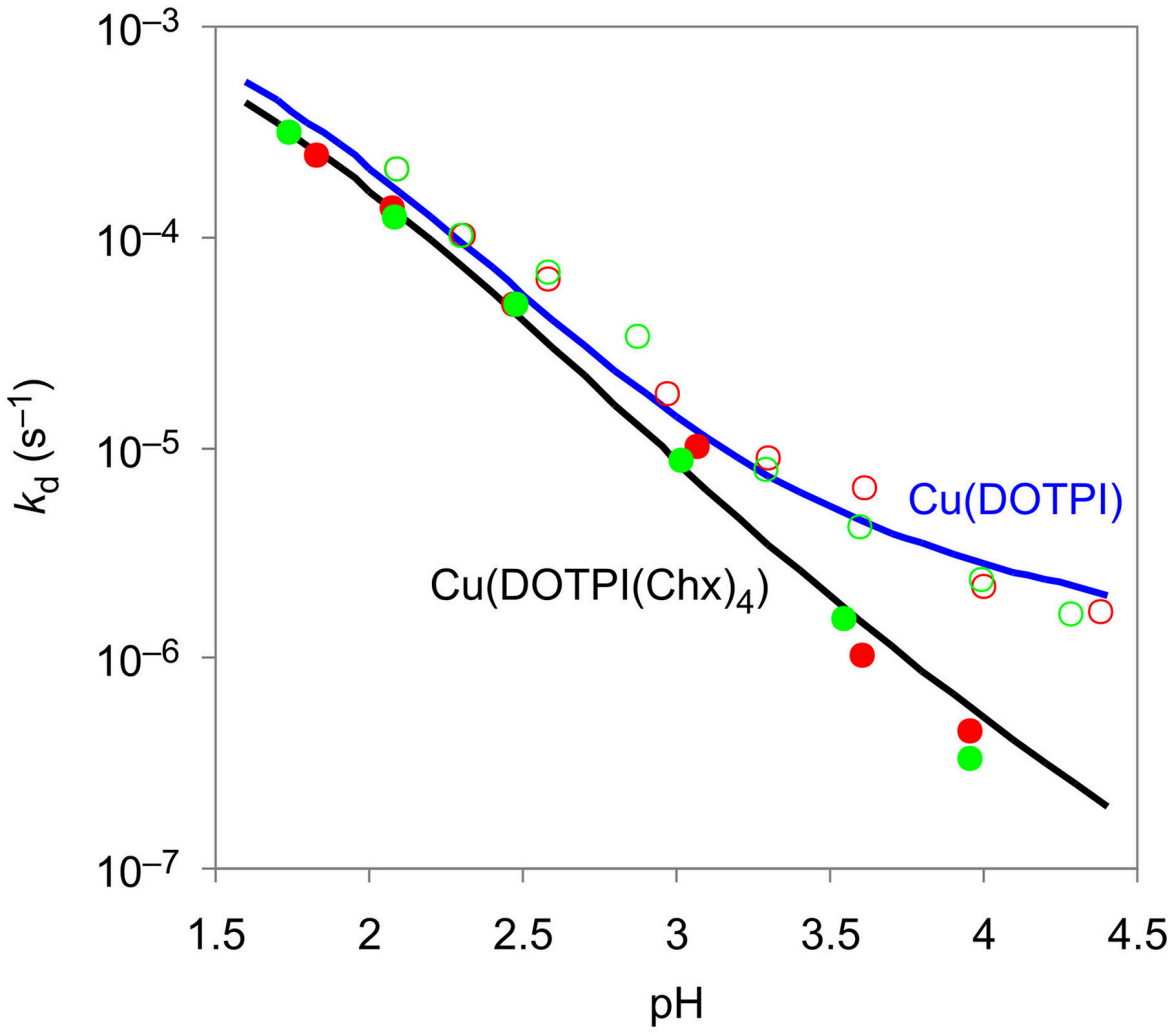
Pseudo-first-order rate constants at 25°C in 0.15 M NaCl for the reaction of Cu^II^ complexes of DOTPI (empty symbols) and DOTPI(Chx)_4_ (filled symbols) with a 20- (red) and 40-fold (green) excess of EDTA at different pH values. Respective *k*_d_ functions were calculated from kinetic and equilibrium data.

Figure 2 shows that the obtained pseudo-first-order rate constants do not depend on the excess of EDTA, and increase with decreasing pH. Hence, it can be assumed that the transchelation of Cu(DOTPI) and Cu(DOTPI(Chx)_4_) occurs by initial protonation, followed by spontaneous dissociation of the protonated Cu^II^ complexes as rate-determining step, while irreversibility is granted by scavenging the released Cu^II^ with EDTA. Similar data and mechanistic implications have been found for Cu^II^ complexes of the smaller triphosphinate macrocycle TRAP (Baranyai et al., 2015), underscoring wide applicability of the transchelation approach for demetallation of chelator conjugates after CuAAC coupling (Notni and Wester, 2016).

Table 3 summarizes rate (*k*) and equilibrium constants (*K*) of all possible pathways for dissociation of the protonated species Cu(H_x_DOTPI) and Cu(H_y_DOTPI(Chx)_4_) (x = 3,4,5,6; y = 1,2; for details see Supplementary Information). According to the proposed mechanism, that is, a fast protonation equilibrium followed by a first-order dissociation reaction rendered irreversible by competitor excess, cleavage of the protonated Cu(H_x_L) complex is promoted by transfer of the proton, which is initially located on the pendant arm, to a ring nitrogen, resulting in the most labile intermediate, an off-cage complex (Baranyai et al., 2015). Overall, the proton displaces the Cu^II^ ion from the coordination cage, causing dissociation of the Cu(H_x_L) complex.

**Table 3:** Rate constants (*k*_Cu(HiL)_) and half-lives (*t*_½_ = ln2/*k*_d_) characterizing the dissociation reactions of Cu(DOTPI) and Cu(DOTPI(Chx)_4_) complexes (0.15 M NaCl, 25°C).

	**Cu(DOTPI)**	**Cu(DOTPI(Chx)_4_)**	**Cu(TRAP) (Baranyai et al., 2015)**	**Cu(TRAP(Chx)_3_) (Baranyai et al., 2015)**
*k*_CuL_ (s^−1^)	–	–	–	6 × 10^−7^
*k*_Cu(HL)_ (s^−1^)	–	(1.7 ± 0.3) × 10^−5^	–	2.3 × 10^−3^
*k*_Cu(_H__2_*L*)_ (s^−1^)	–	(2.9 ± 0.4) × 10^−3^	5 × 10^−6^	–
*k*_Cu(_H__3_*L*)_ (s^−1^)	(1.0 ± 0.5) × 10^−6^	–	6.7 × 10^−5^	–
*k*_Cu(_H__4_*L*)_ (s^−1^)	(3.1 ± 0.6) × 10^−6^	–	2.1 × 10^−3^	–
*k*_Cu(_H__5_*L*)_ (s^−1^)	(4.6 ± 0.5) × 10^−5^	–	–	–
*k*_Cu(_H__6_*L*)_ (s^−1^)	(2.0 ± 0.2) × 10^−3^ (*K*_Cu(_H__6_*L*)_ = 17 ± 8)	–	–	–
*k*_d_ (h^−1^) at pH = 3.0	5.0 × 10^−2^	3.1 × 10^−2^	4.4 × 10^−1^	2.4 × 10^−1^
*t*_½_ (h) at pH = 3.0	**13.8**	**22.4**	**1.57**	**2.94**

*Literature data for corresponding TRAP complexes (Baranyai et al., 2015) are included for comparison*.

A comparison of the rate constants (*k*_Cu(_H__i_*L*)_) obtained for protonated species Cu(H_x_DOTPI) and Cu(H_y_DOTPI(Chx)_4_) confirms that dissociation of the uncharged species, [Cu(H_2_DOTPI(Chx)_4_)] and [Cu(H_6_DOTPI)], which likely contains two non-coordinated and protonated phosphinic oxygen atoms, occurs with a very similar rate. Moreover, the dissociation rates of [Cu(H_6_DOTPI)] and [Cu(H_2_DOTPI(Chx)_4_)] are very similar to those of [Cu(H_4_TRAP)] and [Cu(HTRAP(Chx)_3_)] (Baranyai et al., 2015), which might be explained by similar activation parameters characterizing the proton transfer process from the phosphinate oxygen to the ring nitrogen, resulting in comparable dissociation rate constants at pH < 2.

**Figure 3:**
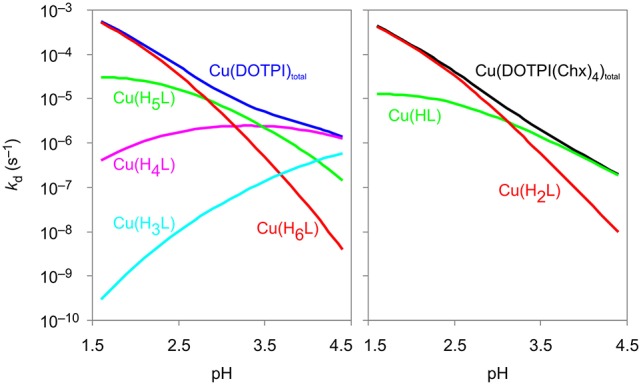
Contributions of Cu(DOTPI) and Cu(DOTPI(Chx)_4_) complexes in different protonation states to the overall rates of the dissociation reaction.

Figure 3 shows the calculated contributions of individual dissociation rates for all protonated Cu(H_x_DOTPI) and Cu(H_y_DOTPI(Chx)_4_) species to the overall demetallation rates (see also Figures S3, S6), which decrease with increasing pH. In the pH range 1.5–4.5, dissociation of Cu(DOTPI) and Cu(DOTPI(Chx)_4_) occurs by the decomplexation of the various protonated species, respectively. In this respect, [Cu(H_6_DOTPI)] and [Cu(H_2_DOTPI(Chx)_4_)] are equivalent as they both carry two phosphinate-bound protons and hence, show similar dissociation rates. Since these species are prevailing at pH < 2, they govern overall dissociation rates which are, therefore, nearly identical (see also Figure 2). On the other hand, at pH > 4, a substantial amount of protonated (and thus, labile) species is observed only for the Cu(DOTPI) system, explaining the higher inertness of Cu(DOTPI(Chx)_4_) complexes.

**Figure 4:**
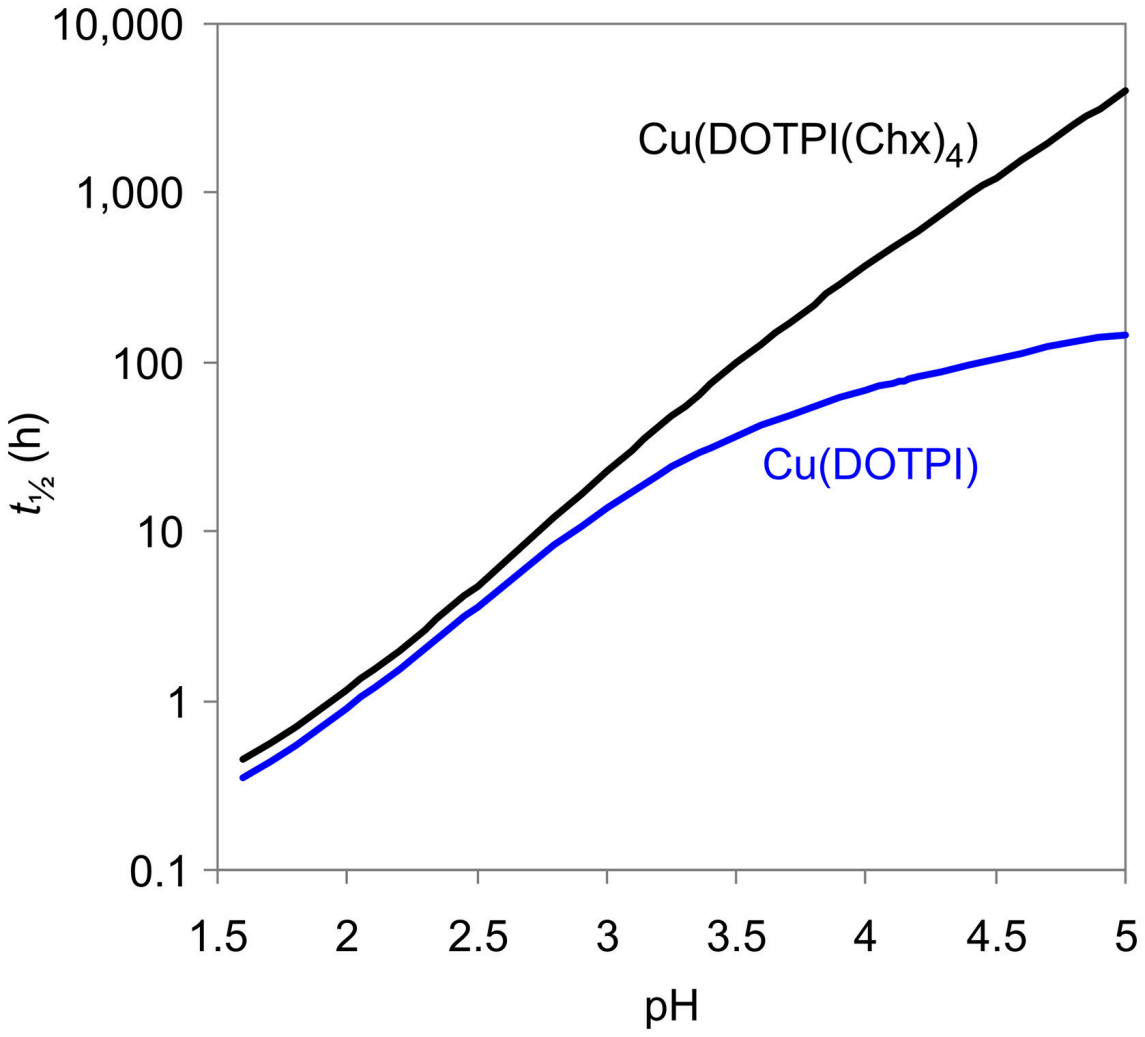
Dissociation half lives (*t*_½_ = ln2/*k*_d_) of Cu(DOTPI) and Cu(DOTPI(Chx)_4_) as functions of pH (25°C, 0.15 M NaCl).

Figure 4 displays the quintessence of the kinetic studies for practical consideration, namely, the dissociation half-lives (*t*_½_) of Cu(DOTPI) and Cu(DOTPI(Chx)_4_) as functions of pH (see Supplemental information, Equation 18). It shows that loss of protonable carboxylates by amide functionalization results in higher *t*_½_ of the respective Cu(DOTPI(Chx)_4_) complexes at higher pH; at pH > 4, the increase exceeds one order of magnitude. On the other hand, Cu^II^ removal from both neat and decorated DOTPI occurs with similar efficiency at lower pH. In view of the calculated *t*_½_, it appears recommendable to carry out such reactions at pH values below 3, preferably at pH = 2, while slightly elevated temperature (e.g., 50°C) will also substantially accelerate the reaction (Baranyai et al., 2015). Apart from that, a *t*_½_ of > 4,000 h for dissociation of Cu(DOTPI(Chx)_4_) at pH > 5 suggests compatibility with the positron emitter ^64^Cu (*T*_½_ = 12.7 h) for application in ^64^Cu-PET imaging.

### Application

**Figure 5:**
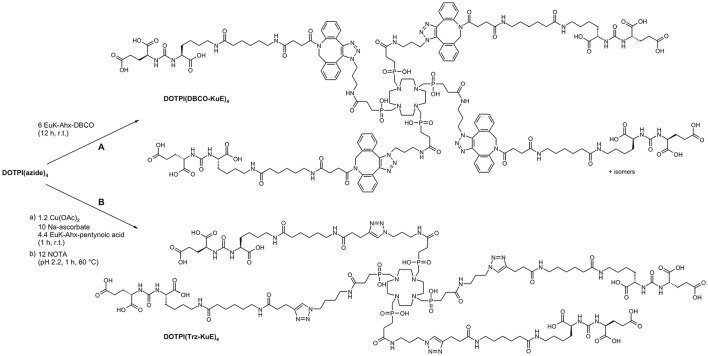
Synthesis of symmetrical tetrameric prostate-specific membrane antigen (PSMA) inhibitor conjugates via DBCO-driven, strain-promoted (SPAAC, path **A**), or Cu^I^-catalyzed (CuAAC, path **B**) alkyne-azide cycloaddition (Huisgen-reaction).

In order to demonstrate utility of the CuAAC/demetallation tandem reaction for assembly of large multimeric bioconjugates and application in the design of therapeutic radiopharmaceuticals, the 4-fold azide-decorated building block DOTPI(azide)_4_ (Wurzer et al., 2018) was reacted with alkyne-functionalized KuE (lysine-urea-glutamic acid, an inhibitor motif for prostate-specific membrane antigen, PSMA, EC 3.4.17.21; synonyms: glutamate carboxypeptidase II, NAALADase (Mesters et al., 2006); a membrane-bound zinc hydrolase which is overexpressed by malignant human prostate cancers). As an alternative, the inhibitor was equipped with dibenzo-azacyclooctyne (DBCO) (Agard et al., 2004) for conjugation via “copper-free click chemistry” (strain-promoted alkyne-azide cycloaddition, SPAAC) according to path **B** in Figure 5. Although this approach circumvents the entire demetallation problem, potentially obviating the above study, the disadvantages of SPAAC are limiting its practical value in the present context. Firstly, reaction rates for SPAAC are several orders of magnitude lower than those of CuAAC, requiring adjustment of reaction conditions, for example, a higher excess of reactants, in order to achieve reasonable yields. Secondly, the isomerism of the formed linker moiety gives rise to a total of 6 stereoisomers of DOTPI(DBCO-KuE)_4_ which are hard to separate, if at all. While the isomers are not likely to exhibit noticeable differences regarding their pharmacodynamics, justifying use of the mixture for *in-vivo* application, such action might nonetheless lead to regulatory issues upon clinical translation. Third, introduction of several large, non-polar linker groups usually affects pharmacokinetics, above all, due to increased plasma protein binding. The overall simplicity of the CuAAC/demetallation approach (Figure 5, path **A**) thus appears preferable for this type of synthetic task, not least because of the known robustness and pharmacokinetic inertness of the 1,3-triazole linkage (Horne et al., 2004, 2009; Bock et al., 2007; Liu et al., 2008; Pedersen and Abell, 2011; Davis et al., 2012; Tischler et al., 2012).

Because the ^177^Lu-labeled tetramers feature nearly identical linker lengths and differ only in the type of linkage, the effect of the dibenzo-azacyclooctane system annulated to the triazole moiety becomes clearly apparent. While PSMA affinities of both tetramers are, as expected (Maschauer et al., 2017), higher than that of clinically applied monomers (Weineisen et al., 2014; Benešová et al., 2016), it is conspicuous that the ^nat^Lu-DOTPI(DBCO-KuE)_4_ exhibits a higher affinity than ^nat^Lu-DOTPI(Trz-KuE)_4_ which is featuring simple 1,2,3-triazole linkages (IC_50_ = 1.4 ± 0.5 vs. 3.4 ± 0.1, respectively). This is because apart from the primary KuE binding site, namely, the catalytic center which contains two hydroxo-bridged Zn^II^ ions, the enzyme PSMA features another hydrophobic site in close proximity (Zhang et al., 2010) which can be addressed by lipophilic groups in the linker (Kularatne et al., 2009; Banerjee et al., 2010). With regard to the DBCO moieties, a virtue is hence made of necessity, because these hydrophobic structure elements contribute to a divalent binding mode. Since the observed increase of affinity is linked to the particular combination of binding sites of the target PSMA, this favorable effect of DBCO cannot be generalized.

**Figure 6:**
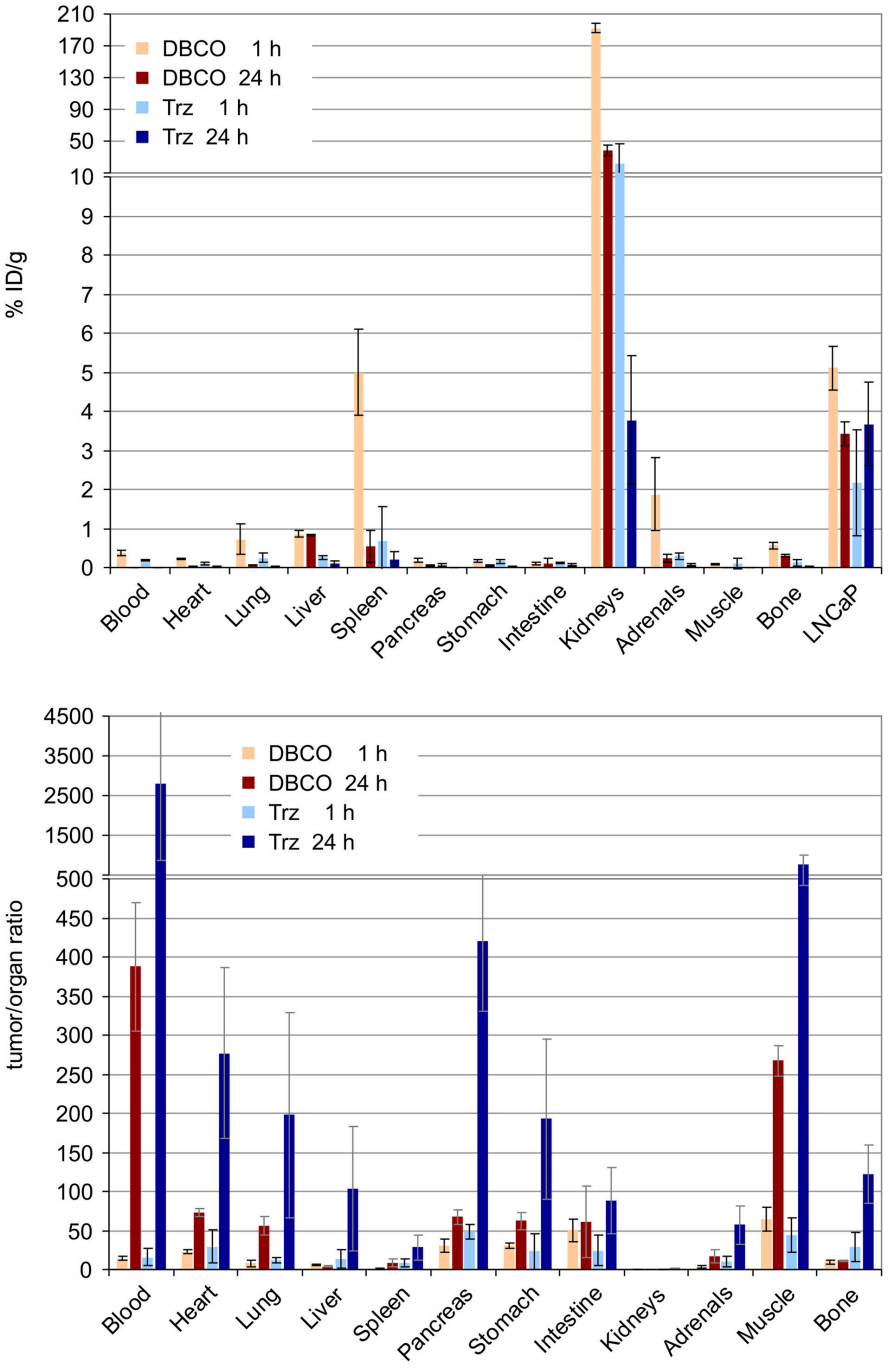
Biodistribution **(Top)** and tumor-to-organ ratios **(Bottom)** for ^177^Lu-DOTPI(DBCO-KuE)_4_ and ^177^Lu-DOTPI(Trz-KuE)_4_ (1.1–4.5 MBq, 0.11–0.15 nmol, *n* = 3), determined 1 and 24 h post injection in SCID mice bearing subcutaneous LNCaP (PSMA-expressing human prostate carcinoma) xenografts.

Apart from that, Figure 6 shows that the higher degree of hydrophilicity of ^177^Lu-DOTPI(Trz-KuE)_4_ (log*D* = −5.0 ± 0.1) results in a remarkably low kidney uptake and a much faster washout from non-target tissues as compared to ^177^Lu-DOTPI(DBCO-KuE)_4_ (log*D* = −4.0 ± 0.1). A virtually similar tumor uptake of both compounds after 24 h gives rise to substantially better tumor-to-organ ratios for ^177^Lu-DOTPI(Trz-KuE)_4_. However, although the acquired data are sufficient for proof-of-principle in the context of this study, a more detailed investigation of the compounds is necessary in order to draw a reliable conclusion regarding their clinical potential.

## Discussion

Replacement of the acetate with amide groups in the pendant arms of DOTPI does not affect the thermodynamic stability (log*K*_ML_ values) of the Ca^II^-, Zn^II^-, and Cu^II^-complexes, while dissociation half-lives of the Cu^II^ chelates at pH > 3 show a clear trend toward higher kinetic inertness. This is another example for the notion that thermodynamic stability constants of complexes do not necessarily correlate with their kinetic inertness, requiring more than just equilibrium data for prediction or confirmation of the suitability of (radio-)metal chelates for *in-vivo* applications. This applies even more because any data acquired under ideal conditions, e.g., in neat solvents or well-defined buffers, should be treated with care in view of a limited transferability to more complex systems, such as living organisms. For example, the aforementioned demetallation half-life of more than 4,000 h for Cu(DOTPI(Chx)_4_) at pH > 5 is no ultimate proof that such conjugates are indeed applicable for ^64^Cu-PET imaging, because other mechanisms than proton-assisted dissociation may contribute to *in-vivo* loss of ^64^Cu, even from highly stable and inert chelates (Bass et al., 2000; Zarschler et al., 2014). A final conclusion on this matter cannot be drawn without further evidence and an *in-vivo* proof of concept, which however is beyond the focus of this study.

## Conclusion

A sufficiently low kinetic inertness of Cu^II^ complexes of amide-functionalized DOTPI derivatives at low pH warrants the practical applicability of the CuAAC/demetallation tandem protocol, which can be conveniently conducted as a one-pot reaction, for the synthesis of symmetrical tetrameric DOTPI conjugates. In view of the inherent limitations of strain-promoted AAC, such as slower reaction kinetics and isomerism/lipophilicity of linkages, CuAAC appears to be the preferable method for this purpose. A pilot *in-vivo* study showed that the DOTPI-based PSMA-targeted radiotherapeutics combine high affinity with excellent clearance from non-target tissues, thus demonstrating the potential of DOTPI as a scaffold for the elaboration of therapeutic radiopharmaceuticals.

## Materials and methods

### Chemical synthesis

#### General

The protected amino acid analogs were purchased from Bachem (Bubendorf, Switzerland) or Iris Biotech (Marktredwitz, Germany). All necessary solvents and other organic reagents were purchased from either, Alfa Aesar (Karlsruhe, Germany), Sigma-Aldrich (Munich, Germany), or VWR (Darmstadt, Germany). The DOTPI chelator (1,4,7,10-tetraazacyclododecane-1,4,7,10-tetrakis[methylene(2-carboxyethylphosphinic acid)]) (Šimeček et al., 2013) and its azide-functionalized derivative DOTPI(azide)_4_ (Wurzer et al., 2018) were synthesized, as described previously. CheMatech (Dijon, France) delivered the NOTA chelator (1,4,7-triazacyclononane-1,4,7-triacetic acid). The PSMA-addressing binding motifs (DBCO-KuE and Trz-KuE) were prepared according to previously published procedures (Wurzer et al., 2018). Analytical and preparative HPLC were performed on Shimadzu gradient systems with a SPD-20A dual wavelength UV/Vis detector (220, 254 nm) with mobile phase gradients combined of purified water (component **A**; from Millipore system) and acetonitrile (component **B**; J.T.Baker®Ultra Gradient HPLC grade, supplemented with 5% H_2_O), both containing 0.1% trifluoroacetic acid. A Nucleosil 100-5 C18 column (125 × 4.6 mm) was used for analytical measurements at a flow rate of 1 mL/min. Preparative HPLC purification was done using a Multospher 100 RP 18-5 μ column (250 × 10 mm) at a flow rate of 5 mL/min. Electrospray ionization (ESI) mass spectra were acquired on a Varian 500-MS Ion Trap spectrometer (Varian, by Agilent Technologies).

#### DOTPI(Chx)_4_

DOTPI·2 H_2_O (85.0 mg, 105 μmol, 1.0 eq) was dissolved in a mixture of anhydrous DMSO (420 μL) and DIPEA (320 μL, 1.89 mmol, 20 eq). Then cyclohexylamine (72.9 mg, 736 μmol, 7.0 eq) and HATU (439 mg, 1.16 mmol, 11 eq) were added in one portion with stirring. After 1 h at room temperature, the red reaction mixture was quenched with water (900 μL). The crude product was purified by size exclusion chromatography (Sephadex G-10 medium, column size: 40 × 3 cm, mobile phase: water, adjusted to pH 3 with HCl), yielding DOTPI(Chx)_4_, as a yellow-green solid (57.7 mg, 53 μmol, 51%). MW (calcd. for C_48_H_92_N_8_O_12_P_4_): 1097.20. HPLC (30–90% **B** in 15 min): *t*_R_ = 13.2 min. ^1^H-NMR (300 MHz, D_2_O, 300 K) δ = 1.13–1.36 (m, 20H), 1.57–1.60 (m, 4H), 1.69–1.74 (m, 8H, C(O)-C**H**_2_), 1.78–1.87 (m, 16H), 2.37–2.45 (m, 8H, P-C**H**_2_-C), 3.13–3.29 (m, 8H, P-C**H**_2_-N), 3.42 (bs, 16H, ring-C**H**_2_), 3.52–3.59 (m, 4H, C(O)-NH-C**H**) ppm. MS (ESI, positive): *m*/*z* = 1098.2 [*M*+H^+^], 1119.9 [*M*+Na^+^], 549.7 [*M*+2 H^+^].

#### DOTPI(DBCO-KuE)_4_

DOTPI(azide)_4_·TFA (2.00 mg, 1.65 μmol, 1.0 eq) and DBCO-KuE (7.13 mg, 9.90 μmol, 6.0 eq) were dissolved in a 1:1 mixture (*v*/*v*, 2.2 mL) of H_2_O and *tert*-butanol and stirred for 12 h at room temperature. Subsequent purification by preparative HPLC yielded DOTPI(DBCO-KuE)_4_·TFA as a colorless solid (6.02 mg, 1.47 μmol, 89%). MW (calcd. for C_184_H_252_N_40_O_52_P_4_): 3980.10. HPLC (10–90% **B** in 15 min): *t*_R_ = 9.2 min. MS (ESI, positive): *m*/*z* = 1327.9 [*M*+3H^+^], 1335.6 [*M*+2H^+^+Na^+^], 1991.4 [*M*+2H^+^].

#### DOTPI(Trz-KuE)_4_

DOTPI(azide)_4_ (6.0 mg, 4.94 μmol, 1.0 eq), sodium ascorbate (97.9 mg, 494 μmol, 100 eq), and Trz-KuE (11.1 mg, 21.7 μmol, 4.4 eq) were dissolved in a 1:1 mixture (*v*/*v*, 2 mL) of H_2_O and *tert*-butanol. After adding copper(II) acetate monohydrate (1.18 mg, 5.93 μmol, 1.2 eq) dissolved in H_2_O (250 μL) a deep blue reaction mixture was formed, which was stirred for 1 h at room temperature. For demetallation, the mixture was diluted to 6 mL with H_2_O, and NOTA (37.6 mg, 124 μmol, 25 eq) was added. After adjusting the pH to 2.2 by adding 1 M HCl, the solution was heated to 60°C for 2 h. Subsequent preparative HPLC-purification afforded DOTPI(Trz-KuE)_4_·TFA as a colorless solid (1.6 mg, 0.49 μmol, 10%). MW (calcd. for C_128_H_216_N_36_O_48_P_4_): 3151.24. HPLC (10–60% **B** in 15 min): *t*_R_ = 6.9 min. MS (ESI, positive): *m*/*z* = 1576.6 [*M*+2H^+^], 1051.2 [*M*+3H^+^], 788.7 [*M*+4H^+^].

### Equilibrium studies (protonation and stability constants)

The chemicals used for the experiments were of the highest analytical grade. CaCl_2_, ZnCl_2_, and CuCl_2_ solutions were prepared from solid MCl_2_ (*Aldrich*; 99.9%). Concentration of CaCl_2_, ZnCl_2_, and CuCl_2_ solutions were determined by complexometric titration with standardized Na_2_H_2_EDTA and xylenol orange (ZnCl_2_), murexid (CuCl_2_), and *Patton & Reeder* (Ca^2+^) as indicators. The concentration of the DOTPI(Chx)_4_, DOTPI, and EDTA was determined by pH-potentiometric titration in the presence and absence of a large (40-fold) excess of CaCl_2_. All the equilibrium measurements were made at constant ionic strength maintained by 0.15 M NaCl at 25°C.

For determining the protonation constants of DOTPI(Chx)_4_ and DOTPI three parallel pH-potentiometric titration were made with 0.2 M NaOH in 0.002 M ligand solutions. The stability and protonation constants of the Ca^II^ and Zn^II^ complexes formed with DOTPI(Chx)_4_ and DOTPI ligands have been determined by direct pH-potentiometric titration made at 1:1 and 2:1 metal to ligand concentration ratios. (the concentration of ligands was 0.002 M). For the calculation of the log*K*_*ML*_ and log*K*_*MLHi*_ values, the mL base—pH data used were obtained in the pH range 1.7–12.0.

Stability constant of Cu(DOTPI) complex was determined by spectrophotometry, studying the Cu^II^-DOTPI systems at the absorption band of Cu^II^-complex at [H^+^] = 0.01– 0.2 M over the wavelength range of 400–800 nm. Concentrations of Cu^II^ and DOTPI were 1 mM. The H^+^ concentration in the samples was adjusted by addition of calculated amounts of 2.0 M HCl, while ionic strength was not constant in these samples. Samples were kept at 25°C for a week. Absorbance values were determined at 9 wavelengths (550, 575, 600, 625, 650, 675, 700, 725, and 750 nm). For calculation of stability and protonation constants of Cu(DOTPI), molar absorptivities of CuCl_2_, and Cu(H_x_L) species (wherein x = 0, 1, 2 …5) were determined by recording the spectra of 1.0 × 10^−3^, 2.0 × 10^−3^ and 3.0 × 10^−3^ M solutions of CuCl_2_ and Cu(DOTPI) in the pH range of 1.7–11.7. The protonation constants of the complexes Cu(DOTPI) and the stability and protonation constants of the dinuclear Cu_2_(DOTPI) complexes were determined by pH-potentiometric titrations, made at 1:1 and 2:1 metal to ligand concentration ratios.

The stability constant of the Cu(DOTPI(Chx)_4_) complex has been determined by spectrophotometry with the use of competition reactions between DOTPI(Chx)_4_ and EDTA for the Cu^2+^ at pH = 5.0. The concentration of Cu(EDTA) was 0.2 mM, while that of the DOTPI(Chx)_4_ was varied between 0.1 and 1.0 mM (5 samples). The samples were kept at 25°C for 2 weeks. The absorbance values and the molar absorptivities of CuCl_2_, Cu(DOTPI(Chx)_4_), and Cu(EDTA) have been determined at 11 wavelength (300, 304, 308, 312, 316, 320, 324, 328, 332, 336, and 340 nm) values between 300 and 340 nm. The molar absorptivities of CuCl_2_, Cu(DOTPI(Chx)_4_), and Cu(EDTA) were deteremined in 0.05, 0.1, 0.2, 0.3, and 0.4 mM solutions. The absorbance and pH values were determined in the samples after the equilibrium was reached (the time needed to reach the equilibria was determined by spectrophotometry). The spectrophotometric measurements were made with the use of 1.0 cm cells with a Cary 1E spectrophotometer at 25°C. The protonation constants of Cu(DOTPI(Chx)_4_) complex were determined by pH-potentiometric titrations at 1:1 metal to ligand molar ratio.

For pH measurements and titrations, a *Metrohm 785 DMP Titrino* titration workstation and a *Metrohm-6.0233.100* combined electrode were used. Equilibrium measurements were carried out at a constant ionic strength (0.15 M NaCl) in 6 mL samples at 25°C. The solutions were stirred, and constantly purged with N_2_. The titrations were performed in a pH range of 1.7–11.7. KH-phthalate (pH = 4.005) and borax (pH = 9.177) buffers were used to calibrate the pH meter. For calculation of [H^+^] from measured pH values, the method proposed by Irving et al. was used (Irving et al., 1967). A 0.01 M HCl solution was titrated with the standardized NaOH solution in the presence of 0.1 M NaCl. Differences between the measured (pH_read_) and calculated pH (–log[H^+^]) values were used to obtain the equilibrium H^+^ concentration from the pH values, measured in the titration experiments. For the equilibrium calculations, the stoichiometric water ionic product (*pK*_*w*_) is also needed to calculate [H^+^] values in basic conditions. The V_NaOH_ – pH_read_ data pairs of the HCl – NaOH titration obtained in the pH range 10.5–12 have been used to calculate the p*K*_w_ value (p*K*_w_ = 13.85). For the calculation of the equilibrium constants the program PSEQUAD (Zekany and Nagypal, 1985) was used.

### Kinetic studies

The rates of the ligand exchange reactions of Cu(DOTPI) and Cu(DOTPI(Chx)_4_) with EDTA ligand were studied by following the dissociation of Cu(DOTPI) and Cu(DOTPI(Chx)_4_) complexes with spectrophotometry at 340 nm, in the pH range 1.7–4.5, in the presence of the 20- and 40-fold excess of EDTA in order to maintain pseudo-first order kinetic conditions. Concentrations of Cu(DOTPI) and Cu(DOTPI(Chx)_4_) were 0.1 mM. Kinetic studies were performed with *Cary 1E* and *Cary 100 Bio* spectrophotometers, using cell holders thermostated to 25°C. The pre-thermostated solutions were mixed in tandem cells (l = 0.874 cm). The ionic strength of the solutions was kept constant at 0.15 M with NaCl. In order to keep the pH values constant, dichloro-acetic acid (DCA) (pH range 1.5–2.5), chloro-acetic acid (MCA) (pH range 2.5–3.5) and 1,4-dimethylpiperazine (DMP) (pH = 3.1–4.5) buffers (0.01 M) were used. Pseudo-first-order rate constants (*k*_d_) were calculated by fitting the absorbance values to the equation

(1) $${\text{A}}_{\text{t}}=({\text{A}}_{\text{0}}-{\text{A}}_{\text{e}}){\text{e}}^{\left(-{\text{k}}_{\text{d}}\text{t}\right)}+{\text{A}}_{\text{e}}$$

wherein A_0_, A_e_, and A_t_ are the absorbance values at the start, at equilibrium and at the time t of the reaction, respectively. The calculation of the kinetic parameters were performed by the fitting of the absorbance–time data pairs with the *Micromath Scientist* computer program (version 2.0, Salt Lake City, UT, USA).

### Radiochemistry

For ^177^Lu-labeling, 10 μL aqueous NH_4_OAc buffer (1 M, pH = 5.9) were added to 1.0 nmol of the labeling precursor (1 mM in DMSO), 10–40 MBq ^177^LuCl_3_ (Specific Activity > 3,000 GBq/mg, 740 MBq/mL, 0.04 M HCl, ITG, Garching, Germany) and finally filled up to 100 μL with H_2_O. The reaction mixture was heated for 30 min at 95°C and the radiochemical purity was determined using radio-TLC (Silica gel 60, mobile phase: 1:1 mixture of 1 M aqueous ammonium acetate and DMF).

### *In vitro* and *in vivo* evaluation

#### Determination of PSMA affinities

PSMA-expressing LNCaP (human prostate carcinoma) cells were grown in Dublecco modified Eagle medium/Nutrition Mixture F-12 with Glutamax-I (1:1) (Invitrogen), supplemented with 10% fetal calf serum and maintained at 37°C in a humidified 5% CO_2_ atmosphere. For determination of the PSMA affinity (IC_50_), cells were harvested 24 ± 2 h before the experiment and seeded in 24-well plates (1.5 × 10^5^ cells per 1 mL well). The competitive binding assay was carried out as described previously, using the radioiodinated PSMA ligand (^125^I-BA)KuE (Weineisen et al., 2014).

#### Octanol-water distribution coefficients

Approximately 1 MBq of the ^177^Lu-labeled tracer was added to a mixture of 0.5 mL phosphate buffered saline (PBS, pH 7.4) and 0.5 mL *n*-octanol in an Eppendorf tube (*n* = 6). After vigorous mixing of the suspension for 3 min, the vial was centrifuged at 11.500 g for 3 min for phase separation. 200 μL aliquots of each phase were withdrawn and measured in a gamma counter.

#### Biodistribution

The experiments were carried out in accordance with the German Animal Welfare Act (Tierschutzgesetz), and were previously approved by the responsible authority (Regierung von Oberbayern). The animal model, male CB-17 SCID mice bearing subcutaneous LNCaP tumor xenografts, were generated as described before (Weineisen et al., 2014). Approximately 1–4 MBq (varying molar activities; absolute molar amounts of active compound ranging from 0.11 to 0.15 nmol) of the ^177^Lu-labeled PSMA inhibitors were injected into the tail vein of the animals, which were sacrificed 1 and 24 h post injection (*n* = 3 per tracer per time point). Selected organs were removed, weighted and the activities contained were measured in a γ-counter.

## Author contributions

AW: Performed chemical synthesis, radiochemistry, and *in-vivo* studies; AV and DH: Performed the equilibrium and kinetic measurements; FF, FK, and H-JW: Performed interpretation of data and critically reviewed the manuscript; JN: Conceived the study, interpreted the data, and wrote the manuscript. All authors approved the final version of the manuscript.

### Conflict of interest statement

The handling editor declared a past co-authorship with several of the authors, AV, H-JW, JN. The other authors declare that the research was conducted in the absence of any commercial or financial relationships that could be construed as a potential conflict of interest.
