# Supplementary material for: Synthesis of Symmetrical Tetrameric Conjugates of the Radiolanthanide Chelator DOTPI for Application in Endoradiotherapy by Means of Click Chemistry
Source: Front Chem. 2018 Apr 10;6:107. doi: 10.3389/fchem.2018.00107 (PMC5902495; doi:10.3389/fchem.2018.00107)
Supplement: Supplementary file 1 [file DataSheet1.PDF]

1. Mass Spectra

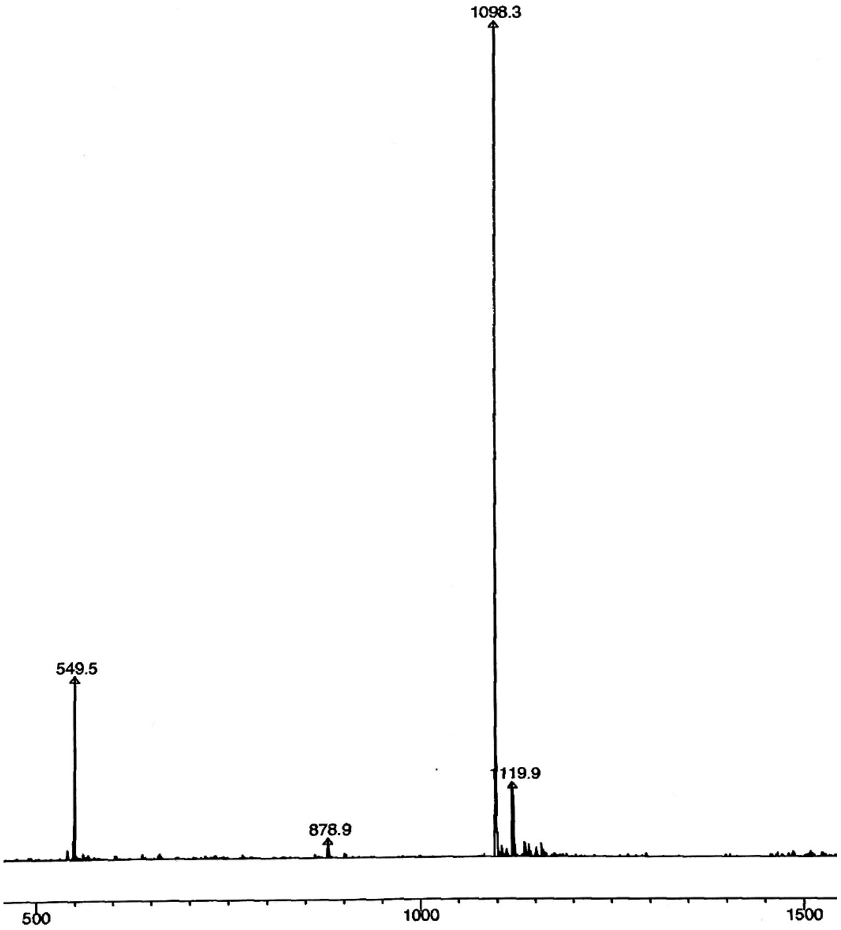

Figure S1: ESI-MS (positive mode) for DOTPI(Chx)$_4$.

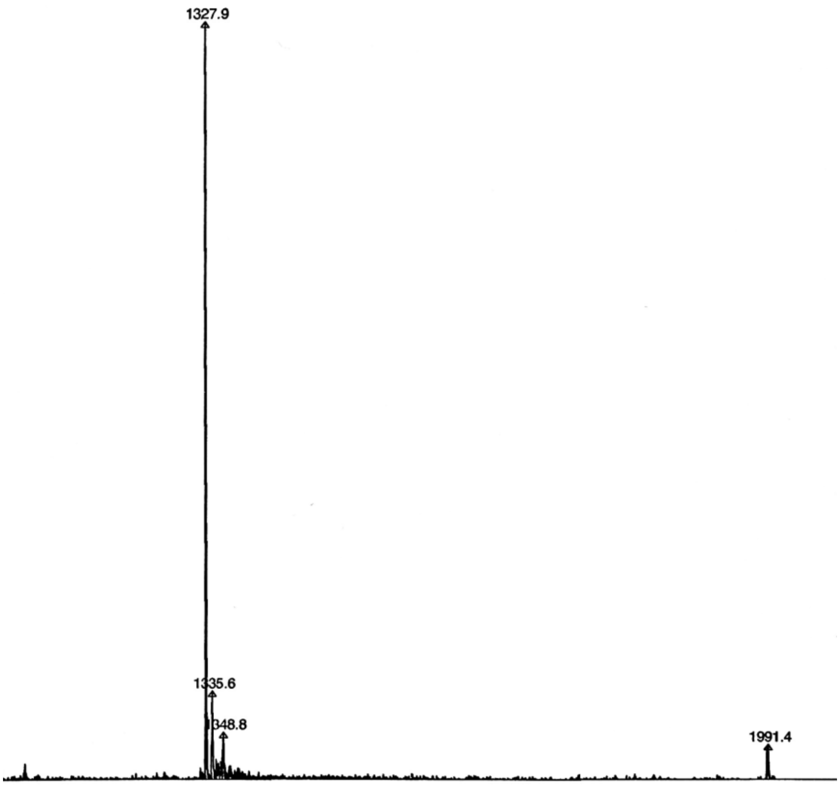

Figure S2: ESI-MS (positive mode) for DOTPI(DBCO-KuE)$_4$.

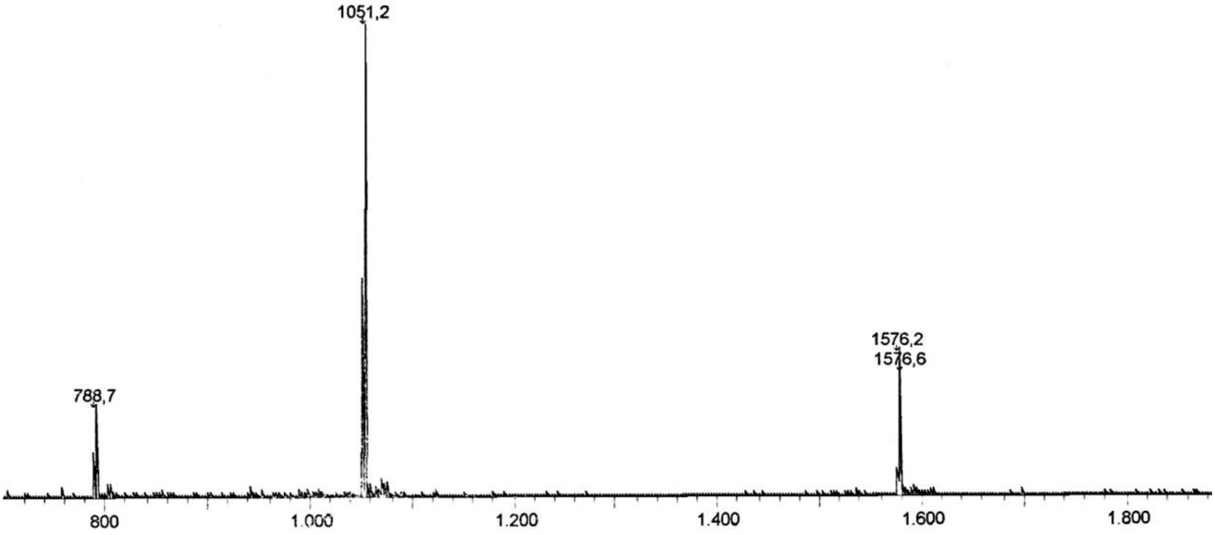

Figure S3: ESI-MS (positive mode) for DOTPI(Trz-KuE)$_4$.

2. Protonation and complexation equilibria of DOTPI and DOTPI(Chx)$_4$ ligands

The protonation processes of the DOTPI and DOTPI(Chx)$_4$, ligands have been studied by pH-potentiometry. The protonation constants ($\log K_i^H$) of ligands are defined according to Eq. (1).

$$K_i^H = \frac{[H_iL]}{[H_{i-1}L][H^+]} \qquad i=0, 1, 2\ldots8 \qquad (1)$$

The stability and protonation constants of DOTPI and DOTPI(Chx)$_4$ complexes with several metal ions were determined by pH-potentiometric and UV/Vis spectrophotometric, methods. The stability and protonation constants of the metal complexes formed with the DOTPI and DOTPI(Chx)$_4$, ligands, defined by Eqs. (2) and (3), are listed in Table S1:

$$K_{ML} = \frac{[ML]}{[M][L]} \qquad (2)$$

$$K_{MH_iL} = \frac{[MH_iL]}{[MH_{i-1}L][H^+]} \qquad (3)$$

where i = 1, 2, 3…5. The K_{ML} and K_{MHiL} values characterizing the formation of DOTPI and DOTPI(Chx)$_4$ complexes of Ca^{II} and Zn^{II} have been calculated from the pH-potentiometric titration data obtained at 1:1 metal to ligand concentration ratios. The protonation constants of the complexes Cu(DOTPI) and Cu(DOTPI(Chx)$_4$) were also determined by pH-potentiometric titrations, made at 1:1 metal to ligand concentration ratio. The pH-potentiometric titrations of DOTPI ligand were also performed at 2:1 metal-to-ligand ratio in order to examine the possible formation of dinuclear Ca^{II}-, Zn^{II}-, and Cu^{II}-DOTPI complexes. The stability and protonation constants of the dinuclear complexes formed with the DOTPI ligand, defined by Eqs. (4) and (5), are listed in Table S1.

$$K_{M_2L} = \frac{[M_2L]}{[ML][M]} \qquad (4)$$

$$K_{M_2LH_i} = \frac{[M_2LH_i]}{[M_2LH_{i-1}][H^+]} \qquad (5)$$

where i=1 and 2. In calculating the equilibrium constants, the best fit of the mL base – pH data was obtained by assuming the formation of ML, MHL, MH_2L, MH_3L, MH_4L, MH_5L, M_2L, M_2LH, and M_2LH_2 complexes with DOTPI and DOTPI(Chx)$_4$.

Stability and protonation constants of Cu(DOTPI) complex have been determined by following the equilibrium reaction (6) with spectrophotometry in the $[H^+]$ range of 0.01–0.2 M, where formation of Cu^{II}, CuH_xL, and H_yL species was assumed (DOTPI: x = 4 and 5; y = 6, 7 and 8).

$Cu^{2+} + H_yL$ **Required parameters are missing or incorrect.** $[Cu(H_xL)] + y\text{-}xH^+$ *(6)*

Some characteristic absorption spectra Cu^{II}-DOTPI systems are shown in Figure S1.

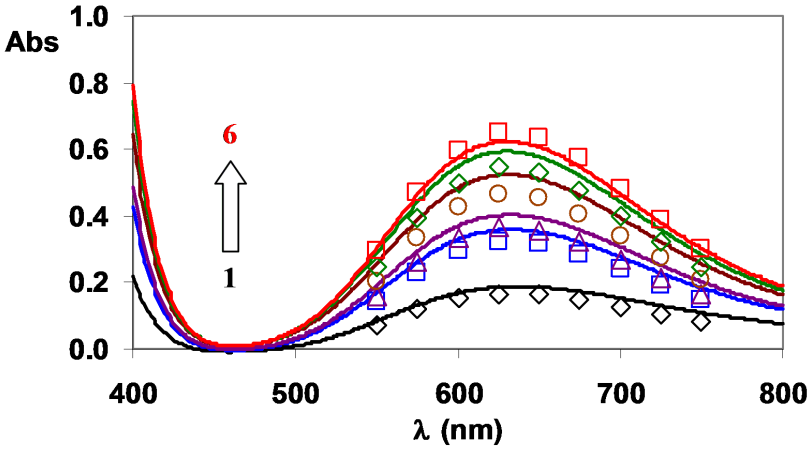

Figure S4: VIS spectra of the Cu^{II} - DOTPI systems. The solid lines and the open symbols represent the experimental and the calculated absorbance values, respectively. ($[Cu^{II}]$=[DOTPI]=1.0 mM, $[H^+]$=0.20 M (**1**), 0.10 M (**2**), 0.081 M (**3**), 0.040 M (**4**), 0.020 M (**5**) and 0.010 M (**6**), $[H^+]+[Na^+]$=0.15 M in the last five samples, 25°C)

Figure S1 show that absorbance values increase with decrease of $[H^+]$. Since positions of absorption maxima and molar absorptivities of $Cu(H_xL)$ and Cu^{II} differ considerably (e.g. Cu^{II}: λ_{max} = 800 nm, ε_{800nm}= 13.45 $cm^{-1} M^{-1}$; $[Cu(H_5DOTPI)]$: λ_{max} = 625 nm, ε_{655nm} = 883.4 $cm^{-1} M^{-1}$), the increase of the absorbance values can be interpreted by the formation of $Cu(H_xL)$ complexes in the $-\log[H^+]$ range 0.71 – 2.25. The stability constants of the CuL complexes have been calculated from the $[H^+]$ and absorbance values obtained at 9 wavelengths between 550 nm and 750 nm, by taking into account the protonation constants of Cu(DOTPI) complex which were determined by pH-potentiometric titration of complexes (Table S1). For calculations, the molar absorptivities of Cu^{II}, $Cu(H_xDOTPI)$ and Cu(DOTPI)

species have been determined at the same 9 wavelengths in separate experiments. The stability constants of [CuL] complexes are presented in Table S1.

In order to get an insight into solution structures of Cu(DOTPI) complexes in different protonation state, the molar absorptivities of Cu^{II}, CuL and Cu(H_xL) species were analysed in detail. Molar absorptivities of the Cu(DOTPI) complexes and the species distribution of the Cu^{II}-DOTPI system are presented in Figures S2 and S3.

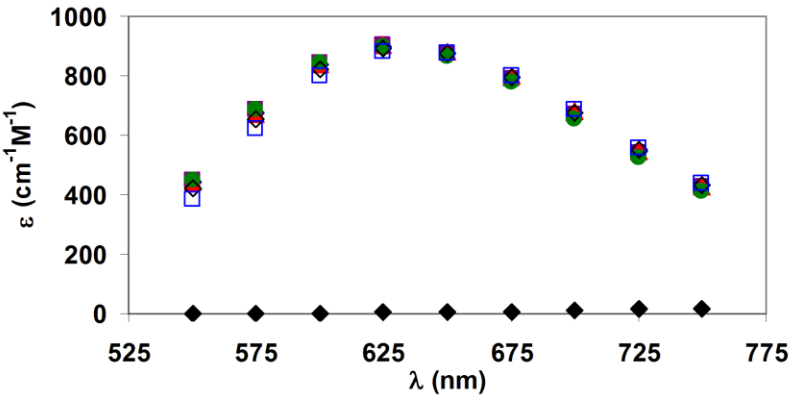

Figure S5: Molar absorptivities of Cu^{II} (◆), Cu(DOTPI) (◆), Cu(HDOTPI) (■), Cu(H_2DOTPI) (▲), Cu(H_3DOTPI) (●), Cu(H_4DOTPI) (◇) and Cu(H_5DOTPI) (□) complexes (0.15 M NaCl, 25 °C)

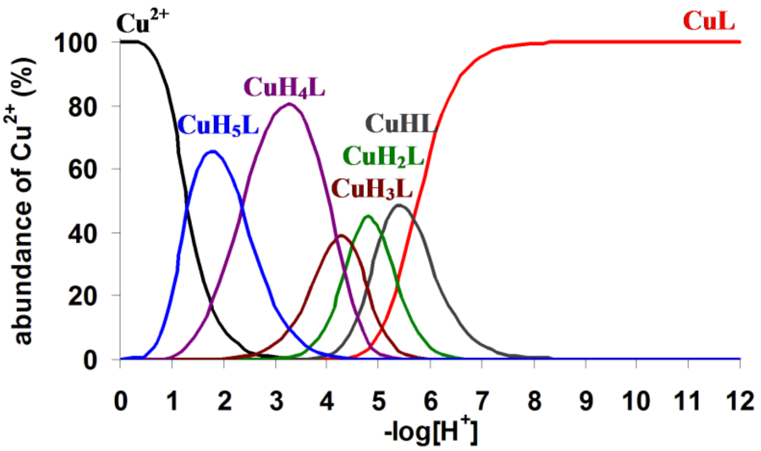

Figure S6: Species distribution of the Cu^{II} - DOTPI system ([Cu^{II}]=[DOTPI]=1.0 mM, 0.15 M NaCl, 25°C)

Figure S2 shows that the maximum of the absorption band is unchanged upon deprotonation of Cu(H_xDOTPI) (x=1, 2, …5) and formation of Cu(DOTPI) complex. Slight incease of the ε values can be identified by the deprotonation of Cu(H_5DOTPI) and the formation of Cu(H_4DOTPI) in wavelength range λ=550 – 625 nm. Because of the similarities, it can be assumed that the structure of Cu(DOTA) and Cu(DOTPI) is comparable in which the Cu^{II} ion

is coordinated by four nitrogen atoms and two acetate or two phosphonate oxygen atoms to give a distorted octahedral ($N_2O_2(N^{ax})_2$) environment, whereas two carboxylate or two phosphinate oxygen atoms are not coordinated, respectively.[1] Based on the very similar molar absorptivities of the Cu(H_xDOTPI) (x=1, 2, …5) and Cu(DOTPI) complexes, it can be assumed that the protonation of the Cu(DOTPI) complex takes place at the non-coordinated carboxylate (Cu(HL), Cu(H_2L), Cu(H_3L) and Cu(H_4L) species) and phosphinate oxygen (Cu(H_5L) species) atoms of the pendant arms.

The equilibrium constants, characterizing the complex formation in the Cu^{II} – DOTPI(Chx)$_4$ system have been determined by pH-potentiometric titration at 1:1 and 2:1 metal to ligand concentration ratios. The equilibrium system could be described by the formation of the species CuL, Cu(HL) and Cu(H_2L) and the protonation constants have been calculated from the pH-potentiometrical data. However, the Cu(DOTPI(Chx)$_4$) is highly stable and its formation was practically complete at about pH=2. For determining the logK_{CuL} value we studied the competition reactions between DOTPI(Chx)$_4$ and EDTA for the Cu^{2+} (Eq. (7)) by spectrophotometry in the wavelenght range 300 – 340 nm (H_4EDTA = ethylenediamine-tetraacetic acid). The pH of the samples was 5, where only Cu(DOTPI(Chx)$_4$) and Cu(EDTA)$^{2-}$ were formed, which is indicated by the appearance of the isobestic points at λ=295 nm in the spectra (Figure S4).

$$\mathrm{Cu(DOTPI(Chx)_4)} + \mathrm{H_yEDTA} \rightleftharpoons \mathrm{Cu(EDTA)} + \mathrm{H_2DOTPI(Chx)_4} \qquad (7)$$

where y = 2 and 3. Species distribution of the Cu^{II}-DOTPI(Chx)$_4$-EDTA system is shown in Figure S5. For the calculation of the stability constant of Cu(DOTPI(Chx)$_4$), the absorbance and molar absorptivity values have been determined at 11 different wavelengths. In the calculation of the logK_{CuL} value characterize the formation of Cu(DOTPI(Chx)$_4$), the protonation constants of EDTA and the stability constants of the Cu(EDTA) complex were taken from the literature (EDTA: logK_1^H=9.43, logK_2^H=6.12, logK_3^H=2.85, logK_4^H=2.18 and logK_5^H=1.77; Cu(EDTA): 19.05).[2] The stability and protonation constants obtained for Cu(DOTPI(Chx)$_4$) is shown in Table S1. The species distribution of the Cu^{II}-DOTPI(Chx)$_4$ system are presented in Figures S5.

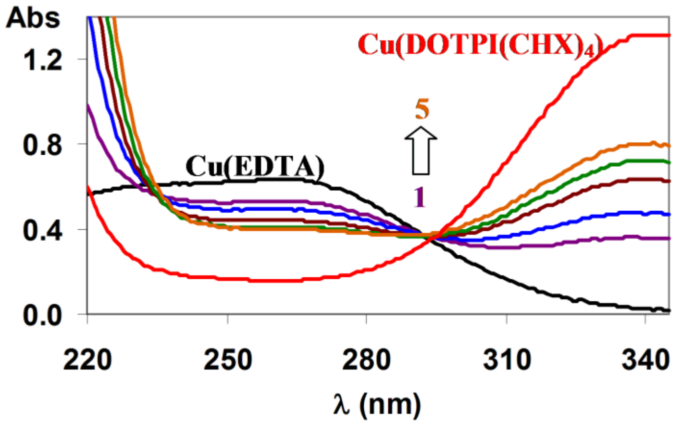

Figure S7: UV spectra of the **Cu(EDTA)**, **Cu(DOTPI(Chx)4)** and Cu^{II} - DOTPI(Chx)4 - EDTA systems ([Cu(EDTA)]=0.2 mM, [Cu(DOTPI(Chx)4)]=0.2 mM, [Cu^{II}]=[EDTA]=0.2 mM, [DOTPI-Chx]= 0.2 mM (**1**), 0.4 mM (**2**), 0.6 mM (**3**), 0.8 mM (**4**) and 1.0 mM (**5**), pH=5.0, 0.15 M NaCl and 25°C)

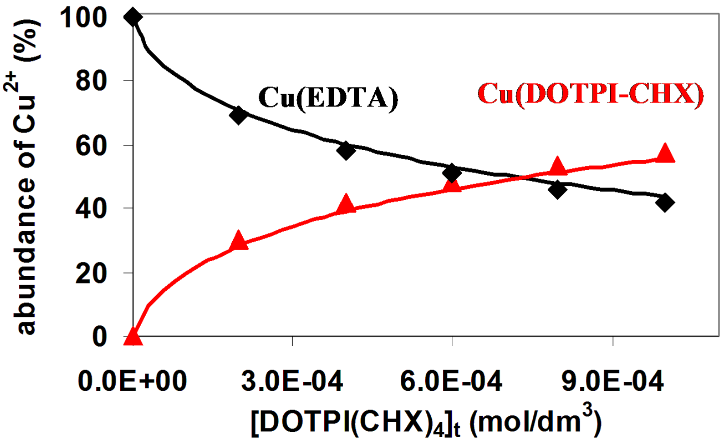

Figure S8: Species distribution of the Cu^{II} - DOTPI(Chx)4 - EDTA system and the relative concentration of Cu(EDTA) (♦) and Cu(DOTPI(Chx)4) (▲) calculated from the UV spectra ([Cu^{II}]=[EDTA]=0.2 mM, pH=5.0, 0.15 M NaCl and 25°C)

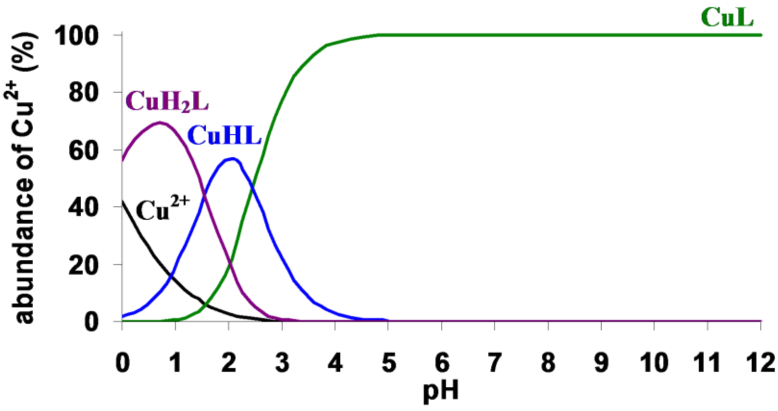

Figure S9: Species distribution of the Cu^{II} - DOTPI(Chx)4 system ([Cu^{II}]=[DOTPI]=1.0 mM, 0.15 M NaCl, 25°C)

Table S1: Stability and protonation constants of Ca^{II}-, Zn^{II}- and Cu^{II}-complexes formed with DOTPI(Chx)$_4$, DOTPI and DOTA ligands (25°C)

	**DOTPI (Chx)$_4$**	**DOTPI**		**$DOTP^{H}$ [3]**	**$DOTP^{Et}$ [4]**	**DOTA [5]**
**I**	**0.15 M NaCl**	**0.15 M NaCl**	**0.1 M Me_4NCl** Error! Bookmark not defined.	**0.1 M KNO_3**	**0.1 M KNO_3**	**0.15 M NaCl**
**CaL**	**8.99 (2)**	**8.65 (3)**	**12.48**	**9.46**	**9.39**	**13.84 (1)**
CaHL	–	6.49 (4)	6.33	3.94	–	3.90 (2)
CaH_2L	–	5.04 (9)	5.96	–	–	–
Ca_2L	–	2.85 (5)	3.20	–	–	–
**ZnL**	**15.13 (1)**	**15.40 (2)**	**18.57**	**14.60**	**15.80**	**17.35 (1)**
ZnHL	2.44 (8)	5.65 (3)	5.37	2.62	–	4.16 (2)
ZnH_2L	–	5.15 (3)	5.09	–	–	3.40 (1)
ZnH_3L	–	4.40 (4)	4.53	–	–	–
ZnH_4L	–	4.42 (1)	3.97	–	–	–
Zn_2L	–	3.78 (4)	3.86	–	–	–
Zn_2LH	–	5.02 (5)	4.96	–	–	–
Zn_2LH_2	–	4.87 (4)	4.51	–	–	–
**CuL**	**20.42 (1)**	**20.30 (5)**	**23.11**	**18.03**	**19.59**	**21.97 (1)**
CuHL	2.46 (1)	5.68 (2)	5.49	–	–	4.08 (1)
CuH_2L	1.55 (2)	5.07 (1)	4.86	–	–	3.41 (1)
CuH_3L	–	4.49 (2)	4.64	–	–	0.83 (2)
CuH_4L	–	4.20 (2)	3.71	–	–	–
CuH_5L	–	2.35 (3)	1.63	–	–	–
Cu_2L	–	4.49 (6)	4.51	–	–	–
Cu_2LH	–	5.04 (3)	4.80	–	–	–
Cu_2LH_2	–	4.71 (5)	4.48	–	–	–

Stability constants of Ca^{II}, Zn^{II} and Cu^{II} complexes formed with DOTPI(Chx)$_4$ and DOTPI (Table S1) are very similar and generally about 2–5 orders of magnitude lower than those of the corresponding complexes of DOTA. The lower stability of the DOTPI(Chx)$_4$ and DOTPI complexes can be explained by lower basicity of the phosphinate oxygens than that of the carboxylate oxygen atoms in the DOTA ligand. Because of the non-coordinating carboxylate groups of the pendant arms, the complexes formed with DOTPI can be protonated at lower pH values via the formation of several protonated M(H_iL) complexes (Table S1). Moreover, these carboxylate groups migth take place in coordination of a second metal ion results in the

formation of dinuclear complexes. Stability constants ($\log K_{M2L}$) of the dinuclear complexes are in the range 2.8 – 4.4 (Table S1), which indicates that just one or two carboxylate groups are coordinated to the second metal ion. On the other hand, the pH-potentiometric studies confirm the formation of mono- and di-protonationated dincuelar Zn_2(DOTPI) and Cu_2(DOTPI) complexes, which confirms that coordination of the second metal ion takes place by one or two carboxylate groups of the pendant arms. For Cu(DOTPI(Chx)$_4$) and Zn(DOTPI(Chx)$_4$) one and two protonation constant/s could be determined, respectively. In these complexes there are probably one and two non-coordinated phosphinate oxygen atoms, which can be protonated at pH values around 1 – 4. Since further deprotonation could not be observed by pH-potentiometry, deprotonation and coordination of the amide groups to Cu^{II}-ion do not take place.

3. Kinetic studies of the ligand exchange reaction of $[Cu(DOTPI(Chx)_4)]$ and Cu(DOTPI) with EDTA

The ligand exchange reactions of $Cu(DOTPI(Chx)_4)$ and Cu(DOTPI) with EDTA (Eq. (7)) were studied by UV-spectrophotometry in the pH range 1.7 – 4.5.

$$Cu(H_xL) \; + \; H_sEDTA \rightleftharpoons Cu(H_zA) + \; H_sL \qquad (7)$$

wherein for $DOTPI(Chx)_4$: x = 0, 1, 2; DOTPI: x = 3, 4, 5 and EDTA: s = 3, 4, 5, z=0, 1, 2). The absorption spectra of the $Cu(DOTPI(Chx)_4)$ + EDTA and Cu(DOTPI) + EDTA reacting systems is shown in Figures S7 and S8.

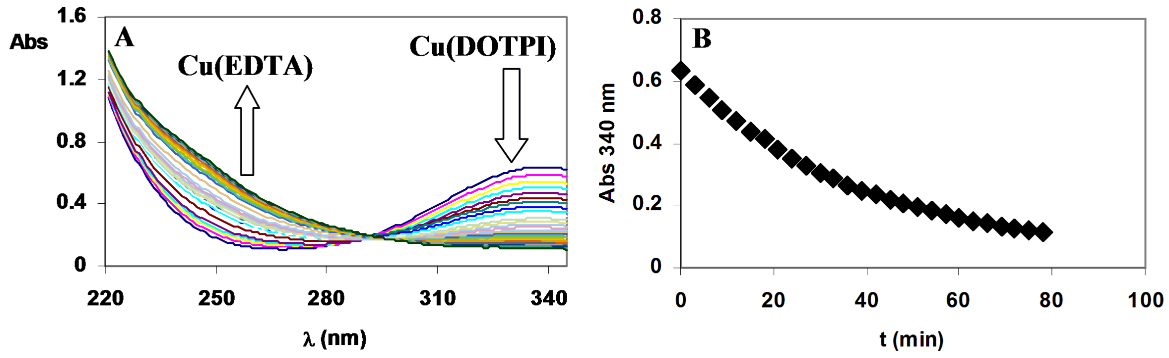

Figure S10: Absorption spectra (A) and kinetic curve (B) of the transmetallation reaction between $[Cu(H_xDOTPI)]$ and H_sEDTA ($[Cu(H_xDOTPI)]$=0.1 mM, [EDTA]=2.0 mM, [DCA]=0.01 M, pH=1.70, 0.15 M NaCl, 25°C)

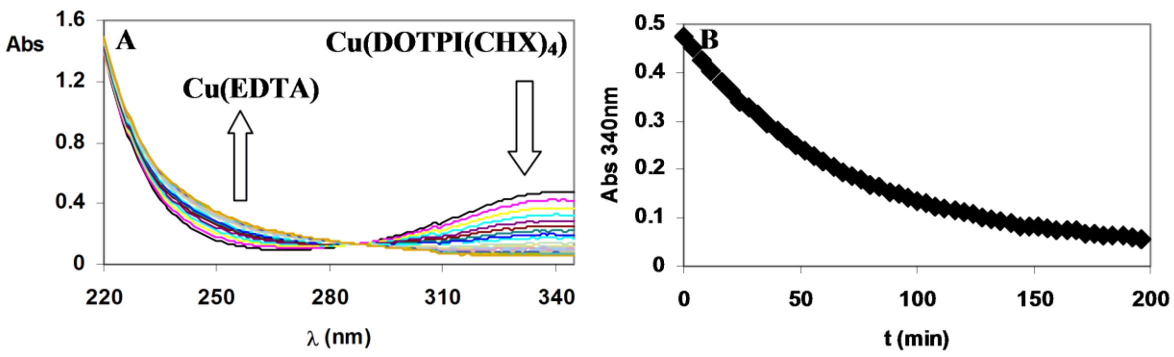

Figure S11: Absorption spectra (A) and kinetic curve (B) of the transmetallation reaction between $[Cu(H_xDOTPI(Chx)_4)]$ and H_sEDTA ($[Cu(H_xDOTPI(Chx)_4)]$=0.1 mM, [EDTA]=2.0 mM, [DCA]=0.01 M, pH=1.80, 0.15 M NaCl, 25°C)

In presence of EDTA excess, the ligand exchange reactions of Cu(DOTPI(Chx)$_4$) and Cu(DOTPI) (Eq. (7) can be treated as pseudo-first-order processes, and reaction rates can be expressed by Eq. (8), wherein k_d is a pseudo-first-order rate constant, and $[CuL]_t$ is the concentrations of the Cu(DOTPI(Chx)$_4$) and Cu(DOTPI) complexes at time *t*.

$$-\frac{d[CuL]_t}{dt} = k_d [CuL]_t \qquad (8)$$

The obtained pseudo-first-order rate constants (k_d) as a function of pH are shown in Figure 1 (main text). Figure 1 shows that the reaction rates (k_d) are independent from the concentration of the competing EDTA ligand. Based on this evidence, it can be assumed that ligand exchange reactions of Cu(DOTPI(Chx)$_4$) and Cu(DOTPI) take place by the dissociation of the complex, followed by a fast reaction between the released Cu^{II} ion and free EDTA ligand. Figure 1 shows that the dissociation rate of Cu(DOTPI(Chx)$_4$) and Cu(DOTPI) complexes increase with the decrease of the pH, which can be interpreted by the protonation of the CuL complexes and the spontaneous dissociation of the protonated Cu(H$_x$L) species. By taking into account the speciation of the Cu(DOTPI) and Cu(DOTPI(Chx)$_4$) in the pH range 1.7 – 4.5 (Figures S3 and S6), the Eq. (8) can be converted into the following form:

$$-\frac{d[CuL]_t}{dt} = \sum k_{Cu(H_iL)} [Cu(H_iL)] \qquad (9)$$

wherein the $k_{Cu(HiL)}$ is the rate constant characterize the dissociation reaction of the Cu(H$_i$L) species (Cu(DOTPI): i=3, 4, 5 and 6; Cu(DOTPI(Chx)$_4$): i=0, 1 and 2). Considering the mass balance of the Cu^{II}-complexes ($[CuL]_{tot}=\Sigma[Cu(H_iL)]$) and the protonation constants of the Cu(DOTPI) and Cu(DOTPI(Chx)$_4$) ($K_{Cu(HiL)}$, Eq. (3) and Table S1), the pseudo-first-order rate constant (k_d) can be expressed as follows:

$$k_d = \frac{\sum k_{Cu(H_iL)} \beta_{Cu(H_iL)} [H^+]^i}{1 + \sum \beta_{Cu(H_iL)} [H^+]^i} \qquad (10)$$

wherein $\beta_{Cu(HiL)}=K_{Cu(HL)} \times K_{Cu(H2L)} \times \ldots \times K_{Cu(HiL)}$. The $k_{Cu(HiL)}$ and $K_{Cu(HiL)}$ values have been calculated by fitting the data points in Figure 1 to Eq. (10). Rate (*k*) and equilibrium (*K*) constants characterizing the ligand exchange reaction of Cu(DOTPI) and Cu(DOTPI(Chx)$_4$) with EDTA are summarized in Table 2. For the direct comparison of the kinetic inertness, the dissociation rates (k_d) and half-lives ($t_{1/2}=\ln 2/k_d$) of Cu(DOTPI) and Cu(DOTPI(Chx)$_4$) were calculated for the pH=3.0 with Eq. (10) by using the rate and protonation constants reported in Table 2.

4. References

1 A. Riesen, M. Zehnder, T. A. Kaden, *Helv. Chim. Acta* **1986**, *69*, 2067–2073.

2 Z. Baranyai, D. Reich, A. Vágner, M. Weineisen, I. Tóth, H. J. Wester, J. Notni *Dalton Trans.*, **2015**, *44*, 11137–11146.

3 K. Bazakas, I. Lukes, *J. Chem. Soc. Dalton Trans.* **1995**, 1133–1137.

4 I. Lázár, A. D. Sherry, R. Ramasamy, E. Brücher, R. Király, *Inorg. Chem.* **1991**, *30*, 5016 –5019.

5 Z. Baranyai, Z. Palinkas, F. Uggeri, E. Brucher, *Eur. J. Inorg. Chem.* **2010**, 1948–1956.
